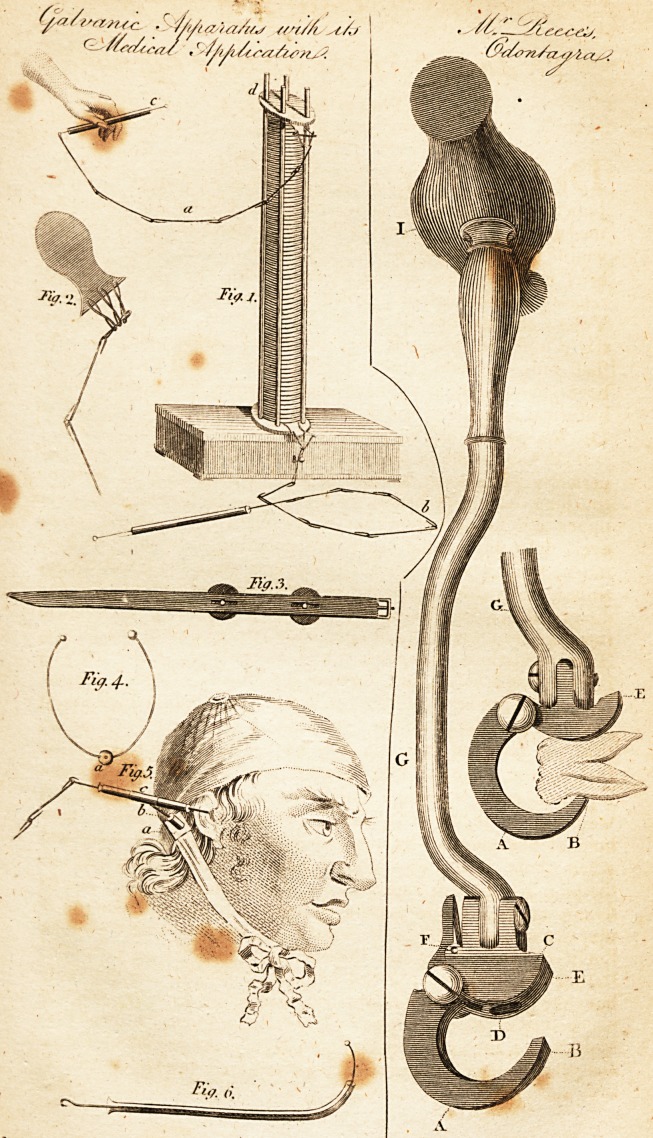# Mr. Reece, on the Extraction of Teeth

**Published:** 1802-03

**Authors:** Richard Reece

**Affiliations:** Henrietta Street, Covent Garden


### *To the Editors of the Medical and Physical Journal.*

GENTLEMEN,

IN compliance with the requeft of your correfpondent, Mr. Cuftance, of Kidderminfter, I have fent you a drawing of the inftrument I took the liberty of recommending to the attention of of the Profeffion, in a former number of your ufeful Journal. Had Mr. C. been induced to have given it a trial, I am confident he would not have required any farther information refpecting it, but have learned from experience " its mode of action," without which I cannot but confider his obfervations rather premature. Thofe gentlemen who are much in the habit of extracting teeth in London have given it the moft decided preference to any other invention; and from my own experience, I am convinced, very defervedly. The plan Mr. Whitford has adopted for the fale of his inftruments is a proof of their having given general fatisfaction; out of two hundred, which he has difpofed of within twelve months, not one has been returned, which was the condition of their fale.

I have alfo fent you a drawing of an inftrument made by Mr. Whitford, from my own inftructions, which, for the fake of diftinction, I have named, " Reece's Odontagra." I fhall briefly notice its peculiarities, and leave your readers to determine as to its merits. Should any of the purchafers of Mr. Whitford's inftrument be inclined to give it a preference, I am defired by Mr. W. to fay, that he will with pleafure exchange it. It differs from Mr. Whitford's in the curvature of the claw (A), the fulcrum (C) being longer, with a depreffion to receive the tooth, and with a fpring (F), to fix the claw. My principal objects to accomplifh in this invention, are, 1ft. To extract the tooth in as perpendicular a direction as poffible, which is nearly effected by the curvature of the claw (A), being more than a femicircle. 2dly. To prevent the breaking of the tooth, and facilitating its extraction, the end of the claw (B) is a little reflected, that it may be introduced between the fcarified gum and tooth, as far down as the alveolar procefs will admit of, and prefs on a greater portion of the tooth. 3dly. To avoid the great pain and injury of the gums from preffure by the ftructure of the fulcrum (C)* in the upper part, which is a concavity to receive the tooth oppofite the upper part of the reflected end of the claw, the preffure of the gum portion (E) does not fall on the contiguous foft parts till the tooth is a little elevated, which afterwards, for its complete expulfion, is very trifling. 4thly. To prevent the claw flipping in the operation by the fpring (F), and which, by taking firm hold of the tooth, fo as to be withdrawn, is fixed between the fulcrum and claw, renders any further ufe of the fcarificator and forceps unneceffary, which fo often alarm the patient with the idea of a fecond operation.

When

* Which rotates on either fide, on the fhank (G).

When the caries of a tooth is gone to that extent, as to leave only a bare ſhell on one ſide, or the whole of the corona deſtroyed, ſo that the remains are level with the gum, the poliſhed part of the fulcrum muſt of courſe reſt on the gum; but from the abſorption of the alveolar proceſs, and the looſe connection of the fangs with the ſocket from the ſame proceſs, the force required for their expulſion is attended with neither pain nor miſchief; and from the formation of the end of the claw, performed with greater eaſe than the uſual way of *punching*.

It is, I think, much to be lamented, that the operation of tooth-drawing ſhould be deemed by the Faculty of ſo little importance, as to be left to the practice of people both ignorant of its nature and ſtructure of the parts, notwithſtanding the frequent occurrences of the ill effects of the rude and unſcientific removal of teeth; beſides, I conceive it often a nice queſtion in ſurgery, to determine whether the extraction of the tooth be abſolutely neceſſary. A caſe ſome time ſince occurred in Monmouthſhire, of a lady who fell a ſacrifice to inflammation ariſing from the irritation of a tooth. Soon after ſhe found it painful, ſhe ſent for a ſurgeon to extract it, which, on account of the contiguous ſoft parts being much inflamed, he poſtponed till the next morning, recommending in the interim, proper means to be taken for its reſolution. In the courſe of the night it had unfortunately increaſed, which on the following morning rendered the operation ſtill more objectionable. On the third morning, the inflammation had extended to the œſophagus and neck, ſo as to prevent deglutition; and on the 5th, it had ſpread to the breaſts, which were amazingly tumefied. About the 9th, a very extenſive ſuppuration took place. About the 11th, it aſſumed a gangrenous appearance, which ſoon terminated in mortification, in ſpite of the efforts of her medical attendants, who are very deſervedly eſteemed gentlemen of great profeſſional judgment and ſkill.

Should the inſtrument I now recommend to the attention of my profeſſional brethren, meet with their ſupport from the reſult of practice, I ſhall be amply rewarded for my trouble by the honour of their approbation.

I have the honour to be, &c.

RICHARD REECE.

*Henrietta Street, Covent Garden.*

*A Caſe*

## Figures and Tables

**Figure f1:**